# Whole structural reconstruction and quantification of epidermal innervation through the suction blister method and skin-clearing technique

**DOI:** 10.1038/s41598-022-16986-7

**Published:** 2022-09-05

**Authors:** Dai Hyun Kim, Se Jeong Lee, June Hoan Kim, Sung Jin Park, Soo Hong Seo, Hyo Hyun Ahn, Woong Sun, Byung-Jo Kim, Im Joo Rhyu

**Affiliations:** 1 Department of Dermatology, Korea University College of Medicine, Seoul, Korea; 2 Department of Anatomy, Korea University College of Medicine, Goryeodae-ro 73 (Anam-dong 5ga), Seongbuk-gu, Seoul, 02841 Korea; 3 Division of Brain Korea 21 Plus Program for Biomedical Science, Korea University College of Medicine, Seoul, Korea; 4 Department of Neurology, Korea University College of Medicine, Seoul, Korea

**Keywords:** Peripheral nervous system, Peripheral neuropathies, 3-D reconstruction

## Abstract

Three-dimensional (3-D) analysis of intraepidermal nerve fibers (IENFs) is conducted to advance assessment methods for peripheral neuropathies and pruritic skin disorders. The skin-clearing technique was proven to be a reliable method for 3-D imaging of IENFs. Nonetheless, it still requires further improvement in the imaging process. The aim of this study was to standardize the 3-D evaluation method of IENFs and to suggest promising 3-D biomarkers for clinical application. A total of nine healthy individuals were prospectively enrolled. The newly adopted suction blister method was combined with the tissue-clearing technique. The detailed structure of the IENFs was reconstructed and quantified using the neuron tracing software. The suction blister method showed improved linear integrity of IENFs compared with those obtained from the previously used salt-split skin test. The 3-D parameters most significantly related to natural aging were the convex hull two-dimensional perimeter and the total length (both *p* = 0.020). The meaningful correlations were followed by total volume (*p* = 0.025), ends (*p* = 0.026), convex hull 3-D surface, and complexity (both *p* = 0.030). Thus, the 3-D parameters could be utilized as possible biomarkers to identify ambiguous pathologies of peripheral neuropathies and pruritic skin disorders.

## Introduction

Three-dimensional (3-D) imaging and quantification of intraepidermal nerve fibers (IENFs) is a promising tool that is used to improve cutaneous nerve biopsy. As such, it has been regarded as a reliable method to assess the status of sensory nerve endings in patients with peripheral neuropathies^1–3^ and to understand dermatologic pathologies including atopic dermatitis^4,5^ and psoriasis^6,7^.

Our previous work reported that the skin clearing and labeling method, cutaneous ACT-PRESTO, was a useful tool for 3-D imaging of IENFs^8^. However, further improvement is still needed in tissue preparation and quantified analysis of 3-D images. The salt-split skin test (SSST), which was previously used for epidermal separation, could cause osmotic damage to delicate internal structures, such as epidermal innervation. The suction blister method generates blister formation by applying negative pressure on the skin. It is an important technique in the surgical intervention of vitiligo^9^ and in the study of epidermal nerves in peripheral nerve disease^10,11^. In this study, the IENFs were expected to be separated from their connection with the underlying subepidermal nerve plexus and to remain intact in the blister roof. These expectations were in contrast to those obtained from SSST.

In our previous study, the 3-D imaging results of IENFs showed the arborized and intermingled nature of epidermal innervation; therefore, it was sometimes difficult to distinguish them from one another^8^. In addition, the 3-D analysis of IENFs was based on the much larger cutaneous volume information rather than on a relatively simple conventional 2-D evaluation based on three randomly selected cross-Sections^1,2^. Therefore, a new approach should be developed to properly quantify true 3-D parameters beyond the number of nerve fibers. The neuron tracing program was adopted for this new workflow since it exhibited its feasibility in assessing complex networks of neurons to sub-cellular dendritic spines and synapses in the field of neuroscience^12–14^. The aim of this study was to advance 3-D imaging of IENFs using suction blisters and to standardize 3-D quantified analysis using a neuron tracing program under normal cutaneous conditions to acquire promising 3-D parameters with clinical significance.

## Results

### Participants

Nine healthy male participants between 26 and 77 years of age were enrolled in the study. The average and median ages were 41.22 ± 15.11 and 37 years, respectively. The biopsy and suction blister method was applied on the left forearm skin in all patients except for one. Since he was a left-handed person, he preferred to use his right forearm for the procedure (Table 1).

**Table 1:** Demographics and quantified intraepidermal nerve fiber densities (IENFDs) of the participants.

Participant number	Age (y)	Sex	Bx. site	IENFD	
				IF (fibers/mm)	Skin clearing (fibers/mm^2^)
1	77	male	Lt. FA	2.43 ± 0.37	165.89
2	36	male	Lt. FA	6.18 ± 0.77	342.61
3	37	male	Lt. FA	3.81 ± 0.52	445.39
4	47	male	Rt. FA	2.96 ± 0.66	322.77
5	44	male	Lt. FA	–	317.36
6	26	male	Lt. FA	7.27 ± 0.41	468.83
7	34	male	Lt. FA	12.20 ± 1.15	780.78
8	28	male	Lt. FA	10.34 ± 0.68	611.28
9	42	male	Lt. FA	16.26 ± 0.12	930.45

*Bx*. Biopsy,* FA* Forearm,* IF* Immunofluorescence.

### Suction blister process

**Figure 1:**
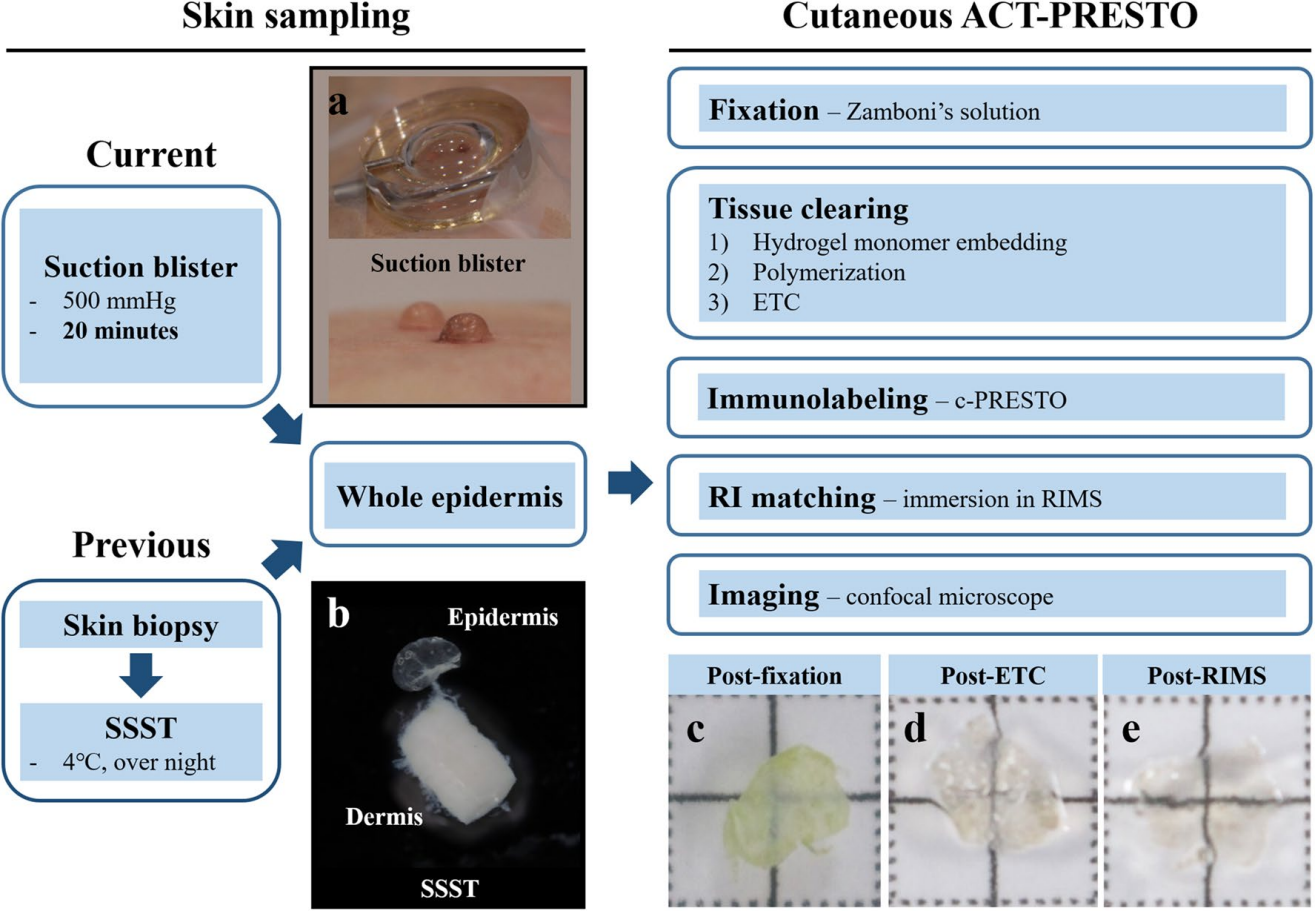
Summarized processes of advanced skin-clearing protocol. The epidermis was separated from the dermis using the (**a**) suction blister method instead of (**b**) a previously adopted SSST^8^. The separated epidermis subsequently proceeded to the cutaneous ACT-PRESTO procedures, including (**c**) fixation, (**d**) electrophoretic tissue clearing (ETC), and (**e**) RI matching. * SSST* Salt-split skin test,* ACT* Active clarity technique,* PRESTO* Pressure-related efficient and stable transfer of macromolecules into organs,* RI* Reflective index,* RIMS* Reflective index matching solution.

The suction blister device was employed to successfully produce complete cutaneous blisters on the participants’ forearms (Fig. 1a) with only minimal and transient pain. The participants experienced no significant side effects associated with the formation of suction blisters. The average time required for this epidermal separation step was 27.20 ± 8.34 min. The blistering time gradually decreased with age. However, the differences were not statistically significant (r = − 0.47, *p* > 0.05).

### Conventional two-dimensional (2-D) assessment

**Figure 2:**
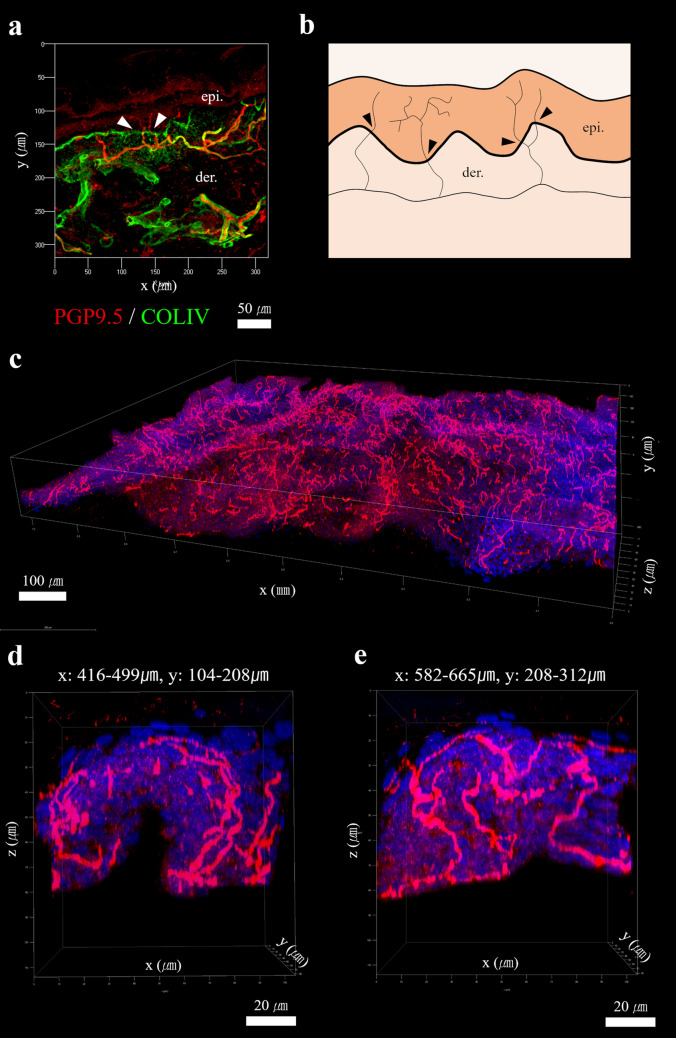
(**a, b**) Conventional two-dimensional (2-D) image of the intraepidermal nerve fibers (IENFs) and counting rules (Reproduced from Eur J Neurol 2005;12:747–758)^1^. (**a**) The punch-biopsied healthy human skin was cryo-sectioned with 80 μm thickness and immunostained with PGP9.5 (red) and type IV collagen (green) to specify cutaneous nerve fibers and dermo-epidermal junction (DEJ), respectively with a Plan-Apochromat 20 × /0.80 M27 lens (z-stack step: 1 μm). Scale bar, 50 μm. (**a, b**) The nerve fibers that definitely crossed the DEJ were regarded as significant data (white and black arrowheads) in quantifying intraepidermal nerve fiber densities (IENFDs). (**c**) Suction blister-based three-dimensional (3-D) image provided true volume information of IENFs in the significantly expanded area of skin (dimensions of x: 1023.51 μm; dimensions of y: 828.96 μm; dimensions of z: 113.50 μm) compared with the previous method, which was based on tissue section. The 3-D images were obtained with a 40 × HC PL APO CS2 40 × /1.30 lens (z-stack step: 0.35 μm). All 3-D images were obtained using 1.28 × zoom with a scan speed of 600 or 700 Hz and merged with multi-frame search mode. Scale bar, 100 μm. (**d, e**) The merged result was subdivided into 64 serial volume images with a confined XY size (x: 83.22 μm; y: 104.13 μm) using LAS X for effective identification of the origin and the course of each nerve fiber. Scale bar, 20 μm.* epi* epidermis,* der* dermis,* PGP9.5* protein gene product 9.5,* COLIV* type IV collagen.

The 2-D assessment of cutaneous nerve fibers was conducted to quantify nerve fibers that vividly crossed the basement membrane of the epidermis (Fig. 2a,b). The average IENF density (IENFD) obtained via two-dimensional assessment of healthy participants was 7.10 ± 4.91 fibers/mm. The IENFDs showed an age-dependent decrease. On the other hand, the degree showed borderline significance (r = − 0.653, *p* = 0.057) (Supplementary Fig. 1).

### ETC and 3-D imaging

The electrophoretic tissue clearing (ETC) process was performed to transform each opaque epidermis obtained from the suction blister into an optically transparent and antibody-penetrable state without substantial architectural changes from the original state. Epidermal separation with suction blisters yielded significantly more intact epidermis with less damage to the internal structural conformation, including linear integrity of nerve fibers, compared with previously reported results obtained from SSST (Supplementary Fig. 2). Therefore, the cutaneous ACT-PRESTO process combined with suction blisters resulted in complete 3-D images of IENFs in large volumes of the epidermis (Fig. 2c, Supplementary Movie 1).

### 3-D reconstruction and morphometric analysis of IENFs

**Figure 3:**
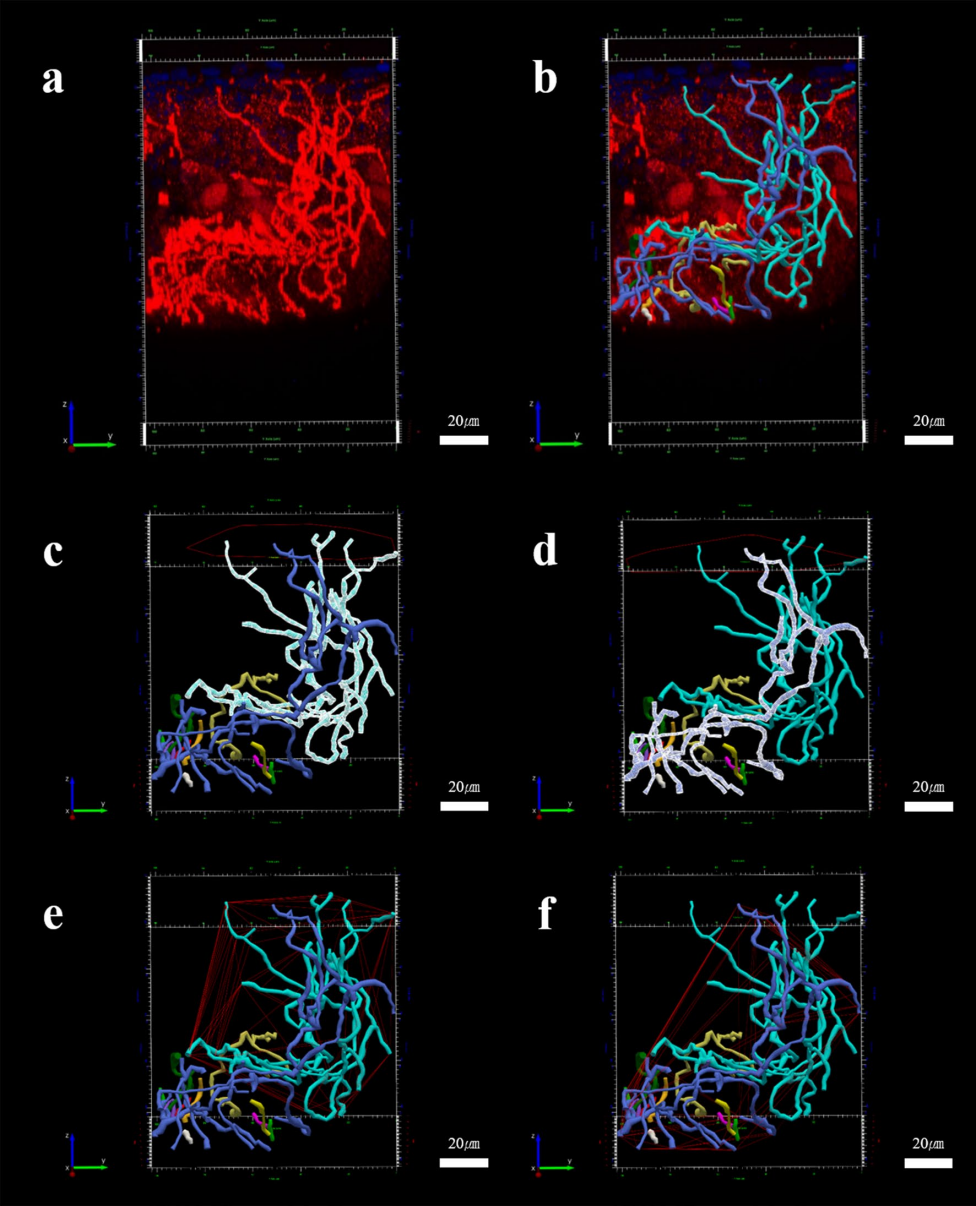
3-D analysis of intraepidermal nerve fibers (IENFs) using software designed for neuronal reconstruction and quantitative analysis, N360. (**a**) The volume information of IENFs obtained from a 34-year-old participant was (**b**) semi-manually reconstructed using the tree tracing with directional kernels method. The reconstructed information was utilized to provide quantitative parameters, including not only conventional numbers of nerve fibers, but also true 3-D data such as nodes, ends, length, volume, complexity, tortuosity, and various angles. The 3-D evaluation also provided the specific field of branched structures controlling a given amount of physical space represented as (**c,d**) convex hull 2-D area and (**e,f**) convex hull 3-D volume. Each IENF, such as the (**c,e**) light blue-colored and (**d,f**) dark blue-colored reconstructed structure was separately analyzed to provide detailed individual morphological changes according to the natural aging process. Scale bar, 20 μm.

The merged volumetric images were divided into 64 same XY-sized 3-D images with sizes of x = 83.22 μm^2^ and y = 104.13 μm (Fig. 2d,e). The XY area of each segmented volume and the whole image was 8665.18 μm^2^ and 0.55 mm^2^, respectively. The cropping of large-scale volume images with even sizes enabled easy recognition of each nerve fiber intermingled with one another and the complex arborization in accordance with the topographical fluctuation of epidermal furrows and ridges (Fig. 2d,e). Every nerve fiber in the given volume was independently traced and reconstructed with different colors using a neuron tracing program (Fig. 3a,b).

The 3-D image tracing and analysis produced numerous quantified parameters, from the well-known Qty and nodes to comparably new ones, such as the ends, total length (μm), total volume (μm^3^), complexity, tortuosity, various angles (°), and results of convex hull analysis. Firstly, the mean value of Qty and Qty/area were 270.22 ± 135.87 fibers and 487.26 ± 244.99 fibers/mm^2^, respectively. There was a significant correlation between the IENFD values quantified using 2-D and 3-D methods, with a correlation coefficient of 0.98 (*p* < 0.01) (Table 1).

In addition, the volume was computed by modeling each piece of each branch as a frustum. The complexity and tortuosity were calculated as follows:

$$ \begin{aligned} Complexity = & \left( {sum\, of \,the\, terminal \, orders + number \, of\, terminals} \right) \\ & \times \left( {total\, dendritic \,length/number \,of \,primary \,dendrites} \right). \\ \end{aligned} $$

$$  Tortuosity = \left( {actual\,length\,of\,segment} \right)/\left( {distance\,between\,the\,endpoints\, of\,the\,segment} \right) $$

The quantified 3-D parameters included various angles (°), such as the planar angle, XY angle, Z angle, and maximum angle. The planar angle pertained to the change in the direction of a segment relative to the previous segment. The XY and Z angles formed spherical coordinate angles at the end of the first segment relative to the start of the next one. The maximum angle was defined only for segments that ended at the nodes. Finally, convex hull analysis was used to measure the size of a neuronal dendritic field. The program was used to measure the size of the dendritic field by interpreting a branched structure as a solid object, thus controlling a given amount of physical space. The amount of physical space was categorized into the convex hull 2-D area (μm^2^), perimeter (μm) (Fig. 3c,d), convex hull 3-D volume (μm^3^), and surface (μm^2^) (Fig. 3e,f).

### Correlation between the 3-D quantified parameters and aging

**Figure 4:**
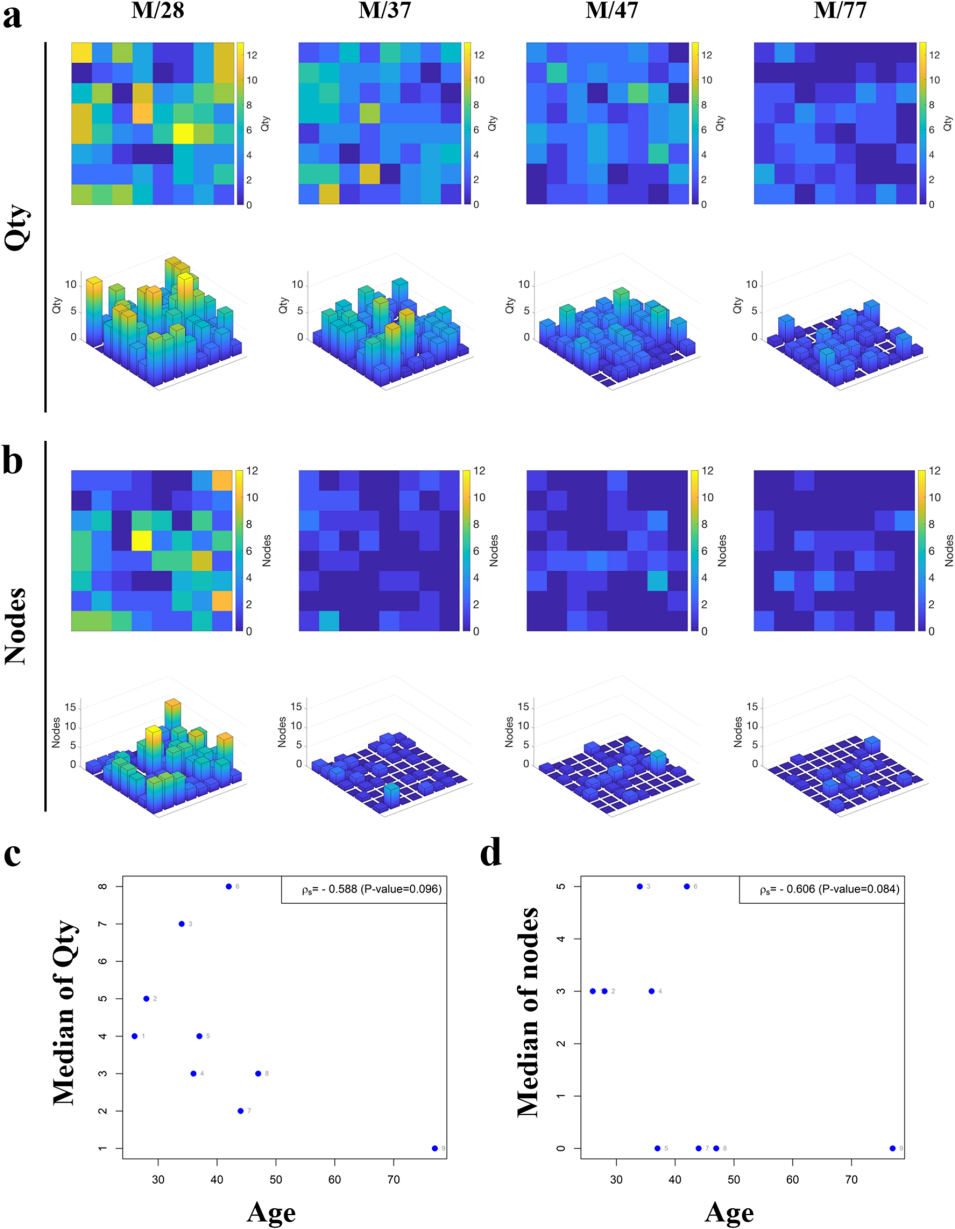
The graphical presentation of age-related changes in previously well-known quantified parameters, (**a,c**) number of nerve fibers (Qty), and (**b,d**) nodes. (**a,b**) Each color in 2-D color maps reflected the degree of quantified results regarding higher values as bright yellow to lower one to dark blue. (**a,b**) The quantified results are presented as 3-D bar graphs, while the height of the bar shows the degree of parameters in each volume.

**Figure 5:**
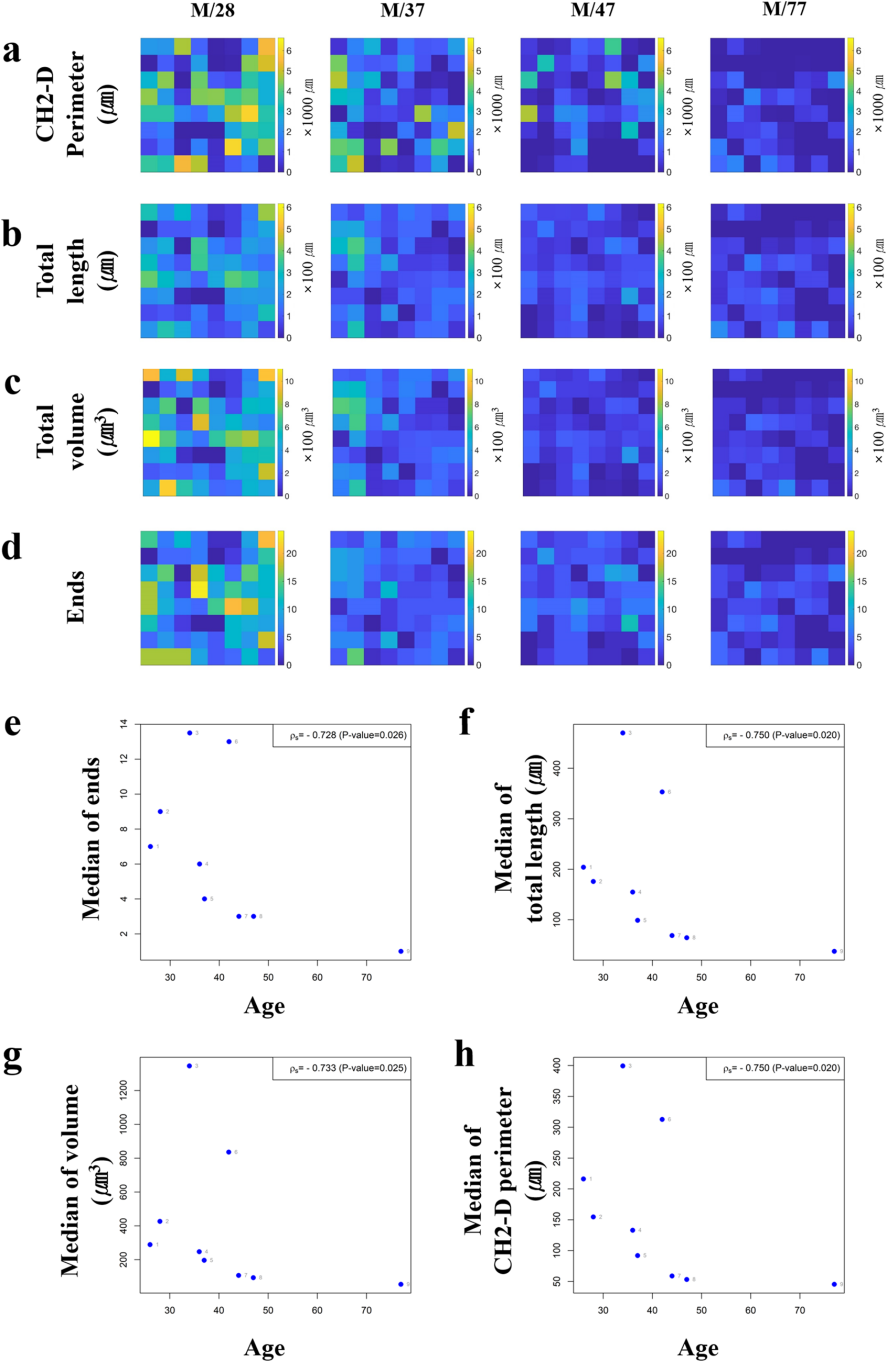
The graphical presentation of age-related changes in the new 3-D parameters, (**a,e**) convex hull 2-D perimeter, (**b,f**) total length, (**c,g**) volume, and (**d,h**) ends that showed the most significant correlations with natural aging. (**a–d**) Each color in 2-D color maps reflects the degree of quantified results regarding higher values as bright yellow to lower one to dark blue.

Well-known parameters, such as the number of nerve fibers, Qty, and nodes, showed borderline significance with aging (r =  − 0.588, *p* = 0.096 and r =  − 0.606, *p* = 0.084, respectively; Fig. 4). The Qty showed a general tendency to decrease and to gradually lose its regional difference according to aging, as represented by color changes, including yellow for high numbers and blue for low numbers (Fig. 4a). As the participants aged, more enlarged cold areas were found in the epidermis. The node also showed an age-dependent decrease. However, it provided a comparably more abrupt loss of its areal difference according to aging and early onset of vast cold areas compared with the patterns of Qty (Fig. 4b). The most statistically significant true 3-D parameters were the convex hull 2-D perimeter and the total length (both r =  − 0.750, *p* = 0.020). The meaningful correlations were followed by total volume (r =  − 0.733, *p* = 0.025), end (r =  − 0.728, *p* = 0.026) (Fig. 5), and complexity (r =  − 0.717, *p* = 0.030). The other convex hull parameters also showed significant correlations with age, including convex hull 3-D surface (r =  − 0.717, *p* = 0.030), 3-D volume, and 2-D area (both r =  − 0.700, *p* = 0.036). Remnant significant correlations with age were observed with tortuosity and XY angle (r =  − 0.667, *p* = 0.050). The majority of angle-related parameters, including planar, max, and Z angles, showed insignificant correlations with age (*p* > 0.05).

Most of the 3-D parameters, except for the Z angle, showed statistically significant correlations with each other (*p* < 0.05, or < 0.01). The age-independent correlations of Qty and the 3-D parameters with significant correlations with age with other parameters were also analyzed to further determine the morphological relationships beyond the influence of natural aging. The Qty showed significant age-independent correlations with the XY angle, ends, tortuosity, length, convex hull 2-D perimeter, volume (*p* < 0.01), planar angle, complexity, max angle, nodes, convex hull 2-D area, 3-D surface (*p* < 0.05) except for the convex hull 3-D volume with borderline significance (*p* = 0.054), and the Z angle with no meaningful correlation (*p* > 0.05). The most significant age-related 3-D parameters, convex hull 2-D perimeter, and length also showed prominent age-independent correlations with most of the 3-D parameters (*p* < 0.01), except for the Z angle (*p* > 0.05). Other highly age-correlated 3-D parameters, ends, complexity, convex hull 2-D area, and volume also displayed consistent age-independent correlations with the 3-D parameters (*p* < 0.05 or < 0.01), except for the Z angle (*p* > 0.05).

## Discussion

Cutaneous nerve biopsy with quantification of IENFD has been regarded as a reliable method for evaluating peripheral neuropathies, including small fiber neuropathy and diabetic neuropathy^1–3^. Cutaneous nerve biopsy has also been adopted for the evaluation of epidermal small-diameter nerve fibers in patients with pruritic skin caused by chronic inflammatory conditions^4–7^. The inflammatory pruritic skin was initially thought to show increased IENFD because of elevated neurotrophic factors, including nerve growth factor produced by activated keratinocytes^15,16^. However, the true status of IENFD in chronic inflammatory pruritic skin remains ambiguous because of conflicting results between elevated^4,6^ and decreased^5,7^ IENFD in the skin of patients with atopic dermatitis and psoriasis. Furthermore, the current cutaneous nerve biopsy only accounts for the number of nerve fibers crossing the basement membrane^1,2^. In addition, it overleaps the complex morphological patterns of branching nerve filaments that lose their connection with the basement membrane during tissue sections. Therefore, a new methodology is required for the advancement of current techniques confined to 2-D tissue section-based analysis that cannot be used to fully elucidate the possible natural or pathological changes in the arborized 3-D nature of epidermal innervation.

The tissue-clearing technique has been developed and utilized to obtain 3-D information in complex biological systems by transforming intact opaque tissue into optically transparent and macromolecule-permeable states without deterioration of the original architecture^17,18^. Our previous work reported that the whole structural 3-D imaging of epidermal innervation was capable of using the tissue clearing and labeling method, ACT-PRESTO, and the quantified IENFDs in 3-D images were meaningfully correlated with the results obtained from conventional cutaneous nerve biopsy^8^. Furthermore, a similar significant correlation between the IENFDs obtained from 2-D and 3-D-based methods was also found in this study (*p* < 0.01, Fig. 2a, c). Therefore, the tissue clearing-based 3-D evaluation of epidermal innervation provides a reliable IENFD dataset comparable to that of the currently used cutaneous nerve biopsy.

The 3-D volumetric analysis that used the tissue-clearing technique yielded definitive expansion in the morphometric analysis of epidermal nerve fibers, and various structural parameters beyond the number of nerve fibers could be quantified in normal or different pathological states. However, further advancement in the procedural and analytical aspects of 3-D imaging is still required for the appropriate and objective quantification of the true 3-D parameters. First, we adopted the suction blister method for epidermal separation, instead of the previously used SSST, to reduce the damage to the delicate structures of IENFs during epidermal separation. The suction blister technique developed by Kennedy et al. was a less invasive method for intact epidermal removal without local anesthesia^1,2,10,11^. The epidermal separation with suction blisters provided comparably more intact morphologic features with consecutive linearity, including small branching segments of each epidermal nerve fiber (Fig. 2c–e) compared with the images obtained from the SSST (Supplementary Fig. 2). The increased imaging quality of epidermal innervation enabled more rapid and objective reconstruction and quantitative analysis because the neuron tracing program could be semi-automatically processed with only minimal subjective intervention, and additional time-consuming adjustments could be avoided when the majority of adjoined backbone points established on PGP9.5 immunostained signals were closely located and sequentially propagated from the base to the end.

The full dendritic drawing of epidermal innervation and quantification was sequentially processed with the neuron tracing and analysis programs, which were often used for the reconstruction of neuronal morphology by tracing, editing, and visualizing image data in 3-D^13,14^. The dendritic segments and spines of hippocampal pyramidal neurons could be traced, modeled, and classified using the neuron tracing software^13^. In addition, the hippocampal and cerebellar microglial and astrocyte morphologies were modeled and compared among various experimental conditions using the 3-D parameters, including the dendritic length, a number of processes, convex data set, and nodes^14^. The accurate reconstruction of complex epidermal innervation requires a neuron tracing software that is fully capable of free rotation and of offering multiple fields of view for the delicate discrimination of arborized epidermal nerve fibers intermingled with one another, as shown in Fig. 3a. The backbone of the dendritic branch was semi-automatically reconstructed using the interactive tree tracing and the directional kernels method, which significantly reduced the investigator’s time and labor. A total of 64 same XY-sized 3-D images required one or two working days on average for complete quantified analysis in our study and the required time tended to increase with a more complex conformation of IENFs of younger subjects. The thickness of the traced nerve fibers was automatically estimated and manifested by the size of the open circles during tracing^13^. The drafted version of the traced dendritic branch should be inspected and edited if necessary to verify that the true configurations are accurately modeled in the right direction and with appropriate thickness, which are sometimes misguided by non-specific noise.

The whole reconstructed epidermal innervation (Fig. 3b) was subsequently processed through a quantified evaluation using the neuron analysis software (Fig. 3c–f). Automated quantification provided comprehensive 3-D information, including total numbers or degree of Qty, nodes, ends, dendritic length, volume, and complexity. Moreover, the individual nerve fibers could be separately quantified, which enabled assessment of the individual characteristics of each intraepidermal nerve fiber. For example, the cyan-colored nerve fibers (Fig. 3c) on the relatively backside and dark blue-colored fibers (Fig. 3d) on the front side were regarded as separate entities originating from different points of the basement membrane, and quantified parameters were individually calculated. Therefore, a separate analysis of the size of a neuronal dendritic field that governs a given amount of epidermal space is possible. The convex hull 2-D (Fig. 3c,d) and 3-D (Fig. 3e,f) entities of each epidermal nerve fiber can be visualized and separately quantified to determine the possible clinical meaning.

Understanding age-related 3-D morphological changes in epidermal innervation is one of the key topics that outstrip the conventional 2-D assessment method, which is capable of showing a simple decrease in the number of nerve fibers according to natural aging, as previously reported^2,19–21^. The young people showed more crowded and branched features of epidermal innervation with higher numbers (Fig. 4a,c) and nodes (Fig. 4b,d) than the older subjects. The intriguing point was that one occurred first, thereby lowering the numbers or simplifying the structures through natural aging. Until now, we are more dependent on the hypothesis that the simplification of the arborized structure, pattern dominant, can be more drastic. In addition, simplification of the arborized structure proceeds first, ‘pattern dominant’ (Fig. 4b,d, Supplementary Fig. 3a), compared to a relatively steady decrease in number, ‘number dominant’ (Fig. 4a,c, Supplementary Fig. 3a). Both Qty and nodes showed borderline significance in the relationship with aging (*p* = 0.096 and *p* = 0.084, respectively). Other quantified 3-D parameters, including convex hull 2-D/3-D data set, length, volume, and ends, displayed more age-sensitive numerical changes (Fig. 5, all *p* < 0.05) compared with Qty and nodes. The epidermal innervation of young participants showed complex morphometry intermingled with one another, and the physically governing area (Fig. 3c,d) and space (Fig. 3e,f) of each nerve fiber naturally overlapped without the blank or dull region of sensation. However, the significant decrease in dendritic governing fields and overlapped space reflected on the convex hull 2-D and 3-D parameters implied that the absolute controlling area and compensating sensory capacity by one another were significantly decreased according to natural aging with the emergence and enlarging anarchy area without the physical reach of epidermal innervation (Supplementary Fig. 3b).

The limitations of the study were the small number of subjects and still prolonged required time from suction blister formation to the 3-D imaging and quantified analysis. A future study with more enlarged subjects is required for not only confirmation of the age-dependent changes of 3-D parameters in our study but also verification of the minimized extent of 3-D images to reduce the excessive time for 3-D imaging and quantification. The required time for blister production could be shortened with firm contact on the skin surface of the device based on our experience. Therefore, the curved region, or hairy area, should be avoided for effective suction blister formation. Lastly, the indispensable requirement of high-cost experimental equipment for the skin-clearing process, confocal microscopy and 3-D quantified analysis could be an entry barrier to impede generalized adoption in the diagnostic evaluation.

In conclusion, reconstruction and quantitative analysis of 3-D epidermal innervation produced sensitive 3-D parameters, including convex hull 2-D perimeter, total length, total volume, and ends. These 3-D parameters seemed to reflect age-dependent morphological changes of IENFs. The scope of the standardized 3-D evaluation tool for epidermal innervation can be broadened in the investigation of unknown or ambiguous structural and functional changes in cutaneous nerve fibers associated with various chronic diseases that are notable for recalcitrant sensory symptoms, such as small fiber neuropathy, diabetic neuropathy, and atopic dermatitis.

## Materials and methods

### Participants

Healthy male participants between 20 and 80 years of age were prospectively recruited from the Korea University Medical Center (KUMC), Anam Hospital. The Institutional Review Board of Korea University Medical Center (KUMC), Anam Hospital, approved the clinical trial (2016AN2056). All participants were fully informed of the possible risks brought about by the procedures in this study. Written informed consent was obtained from all patients. The study was performed in accordance with the Declaration of Helsinki on human subjects. The inclusion criteria were as follows: healthy subjects with no medical history or signs related to peripheral neuropathy, such as diabetes, and a history of receiving chemotherapy. Participants with pruritic cutaneous disorders and a history of undergoing phototherapy were excluded from the study because of the possibility of meaningful alterations in the status of intraepidermal nerve fibers. Dermatologists and neurologists at the KUMC, Anam Hospital, performed clinical and/or neurological examinations.

### Suction blister method and punch biopsy

Two 3-mm suction blisters and one 3-mm punch-biopsied skin parts were obtained from intact forearm skin. Skin blisters were formed by applying a negative pressure of 500 mmHg using a suction capsule device (Aligned Genetics Inc., Anyang, Korea) modified using an apparatus that was kindly provided by Dr. Kennedy^10,11^. The suction capsule with two 3-mm diameter round holes (Fig. 1a) was tightly attached to the surface of the skin, and the area was warmed by exposure to an infrared light device, WHF-312 (Unitec, Seoul, Korea). After confirmation of a full blister, the blister roof was excised using microscissors. Punch biopsies with a size of 3 mm were obtained from patients who were under local anesthesia with lidocaine.

### 2-D assessment

The biopsied specimens were fixed using Zamboni’s solution overnight at 4 °C. The fixed samples were cryosectioned with 80 μm thickness. Three randomly selected sections were immunostained with PGP9.5 (1:800; CEDARLANE) and type IV collagen (1:800; Sigma–Aldrich, St. Louis, MO, USA). A confocal microscope (LSM 700; Carl Zeiss Inc., Jena, Germany) was used for image acquisition with a Plan-Apochromat 20 × /0.80 M27 lens, and serial optical sections taken at intervals of 1 μm were reconstructed using a related software (ZEN; Carl Zeiss Inc.). Quantification of cutaneous nerve fibers was performed using maximum projection images according to the guidelines of the European Federation of Neurological Societies^1,2^.

### Tissue preparation and 3-D imaging

The gently flattened epidermal samples obtained from the suction blister were fixed with Zamboni’s solution (2% paraformaldehyde with picric acid, American MasterTech, CA, USA) overnight at 4 °C. The sample was processed according to the cutaneous ACT-PRESTO protocol (Fig. 1). In summary, the fixed epidermis (Fig. 1c) was processed through ETC under static condition including 1.5 A at 37 °C for 4 h (Fig. 1d). The specific condition for the ETC process was deducted from the previous studies to facilitate complete tissue clearing and prevent unwanted morphological alterations that could be manifested as irreversible shrinkage or significant color change^8^. The cleared epidermis was immunolabelled with PGP9.5 (1:400; CEDARLANE) aided by centrifugal force. The cleared skin was immersed in a solution containing PGP9.5 or Cy3 (1:500; Jackson ImmunoResearch, West Grove, PA, USA) and centrifuged at 800 × g for 3 and 2 h to accelerate penetration of primary and secondary antibodies, respectively. The immunostained tissue was subsequently immersed in a reflective index-matching solution (RIMS) (Fig. 1e). The immunostained cutaneous nerve fibers were imaged by confocal microscopy (SP8; Leica Microsystems, Wetzlar, Germany) using a 40 × HC PL APO CS2 40 × /1.30 oil immersion lens with z-stacking taken at intervals of 0.35 μm. All 3-D images were obtained using 1.28 × zoom with a scan speed of 600 or 700 Hz. A total of 20 volume images with an overall area of 0.83–0.86 mm^2^ in the aspect of the XY-plane and height, varying between 100 and 200 μm, were consecutively imaged and merged by tile scan with fine accuracy and multi-frame search mode. Each volume image was comprised of compiled stacks of approximately 250 to 500 separate images due to optimal z-stacking intervals of 0.35 μm, and a total of 20 volume images requires 10–15 h for complete 3-D imaging and merging.

### 3-D reconstruction and analysis of IENFs

The merged volumetric 3-D images of IENFs aided by image software were divided into 64 identical XY-sized (x: 83.22 μm; y: 104.13 μm) separate volume images using image-processing software (LAS X; Leica Microsystems) to secure visual discrepancy among the complexed nerve fibers that were often mingled with one another. The detailed structure of IENFs was semi-automatically traced and reconstructed using a neuron tracing program, the N360 (Neurolucida 360; MBF Bioscience, Williston, USA), with interactive tree tracing and directional kernel method. The nerve fiber originating from the basement and not the side wall of the volume was regarded as significant and traced. The basal side of the epidermis was readily verified by a more compact cellular composition compared to the upper side represented by nuclear staining. The fragment that lost its connection with the basement was not traced. The evaluators were blinded to the specific information of the participants during the entire process of nerve tracing to prevent possible bias. Particularly, the backbone of the epidermal nerve fibers was traced by designating the starting point in the basement membrane. The semi-automatically estimated continuous backbone path was followed during movement of the cursor along the PGP9.5-immunostained nerve fibers. It schematically showed the inspection of dendritic segment thickness by different backbone diameters^13^. The misplaced backbone points departed from the actual path. Wrongly designated thickness was manually moved and adjusted to the correct location using the appropriate thickness that was confirmed by repetitive 360-degree rotation of the operating volume images. The fully traced images were moved to an N360 Explorer (MBF Bioscience, Williston, USA) for the quantified morphometric analyses. The branched structure analysis and convex hull analysis were the mainstays of the evaluation. Each epidermal nerve fiber in a given volume image was quantified separately, and the results provided true 3-D parameters of epidermal innervation. The quantified parameters of 64 different volumes were also displayed with a 2-D color map and 3-D bar graph using MATLAB.

### Statistical analysis

All statistical analyses were performed using the IBM SPSS, version 25.0, for Windows (IBM Corp., Armonk, NY, USA). Statistical differences between the paired parameters were analyzed using the Wilcoxon signed-rank test. The age-related correlations of each three-dimensional parameter were processed using the Pearson’s or Spearman’s correlation analysis. Statistical significance was set at *p* < 0.05.

## Supplementary Information

Supplementary Information 1.

Supplementary Video 1.

## Data Availability

All data and materials relevant to the study are available from the corresponding author on reasonable request.
