# Supplementary material for: Whole structural reconstruction and quantification of epidermal innervation through the suction blister method and skin-clearing technique
Source: Sci Rep. 2022 Sep 5;12:13596. doi: 10.1038/s41598-022-16986-7 (PMC9445051; doi:10.1038/s41598-022-16986-7)
Supplement: Supplementary file 1 — Supplementary Information 1. [file 41598_2022_16986_MOESM1_ESM.docx]

**SUPPLEMENTARY MATERIALS TO:**

**Whole Structural Reconstruction and Quantification of Epidermal Innervation through the Suction Blister Method and Skin-Clearing Technique**

Dai Hyun Kim^1,2^, Se Jeong Lee^2^, June Hoan Kim^2^, Sung Jin Park^1^, Soo Hong Seo^1^, Hyo Hyun Ahn^1^, Woong Sun^2,3^, Byung-Jo Kim^4^ and Im Joo Rhyu^2,3*^

^1^ Department of Dermatology, Korea University College of Medicine, Seoul, Korea

^2^ Department of Anatomy, Korea University College of Medicine, Seoul, Korea

^3^ Division of Brain Korea 21 Plus Program for Biomedical Science, Korea University College of Medicine, Seoul, Korea

^4^ Department of Neurology, Korea University College of Medicine, Seoul, Korea

*Correspondence: Im Joo Rhyu, MD, PhD

Department of Anatomy, Korea University College of Medicine

Goryeodae-ro 73 (Anam-dong 5ga), Seongbuk-gu, Seoul, 02841, Korea

Tel: +82-2-2286-1150; Fax: +82-2-2286-1387

E-mail: [irhyu@korea.ac.kr](mailto:irhyu@korea.ac.kr)

**Supplementary Figure 1.** The correlation between conventional 2-D based intraepidermal nerve fiber densities (IENFDs) and age (r = -0.653, *p* =0.057).

**
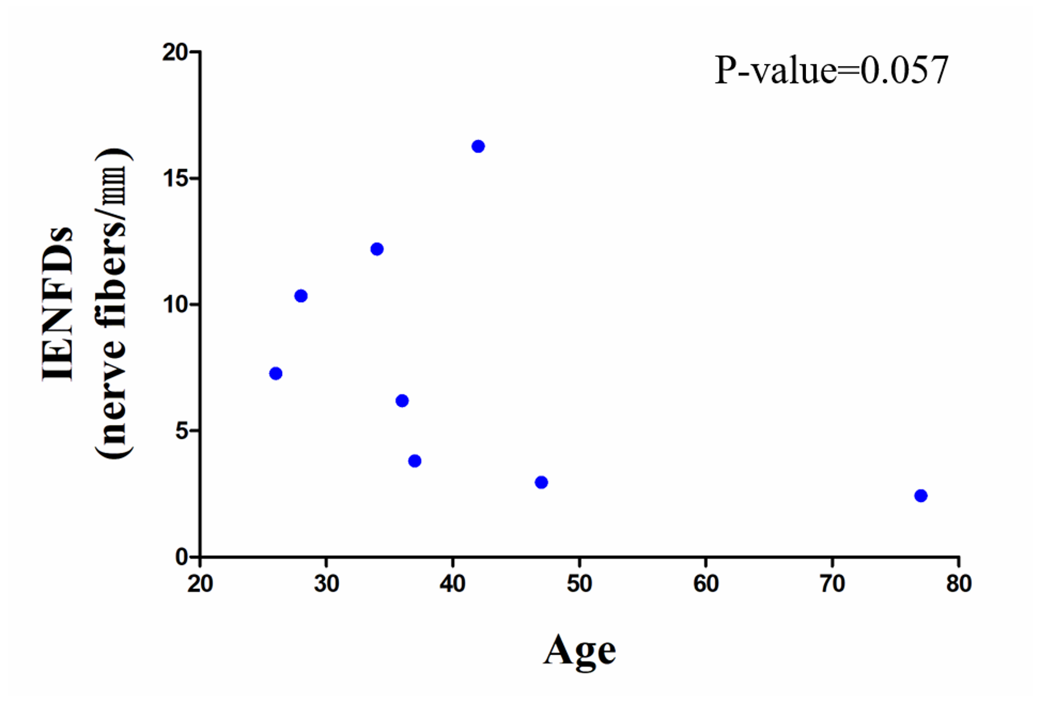
**

**Supplementary Figure 2.** Comparison between images of PGP9.5-labeled (red) intraepidermal nerve fibers obtained from (a, b) SSST and (c, d) suction blister technique. (a, b) The intermittently fragmented appearance found in the formal methodology, SSST, acted as a hurdle in an expedited 3-D analysis. However, (c, d) the comparably more intact structural integrity was imaged through the advancement of the skin sampling method. All 3-D images were obtained using a confocal microscopy with a 40× HC PL APO CS2 40×/1.30 lens (z-stack step: 0.35㎛). Scale bar, 20㎛.

Abbreviations: SSST, salt split skin test; PGP9.5, protein gene product 9.5.


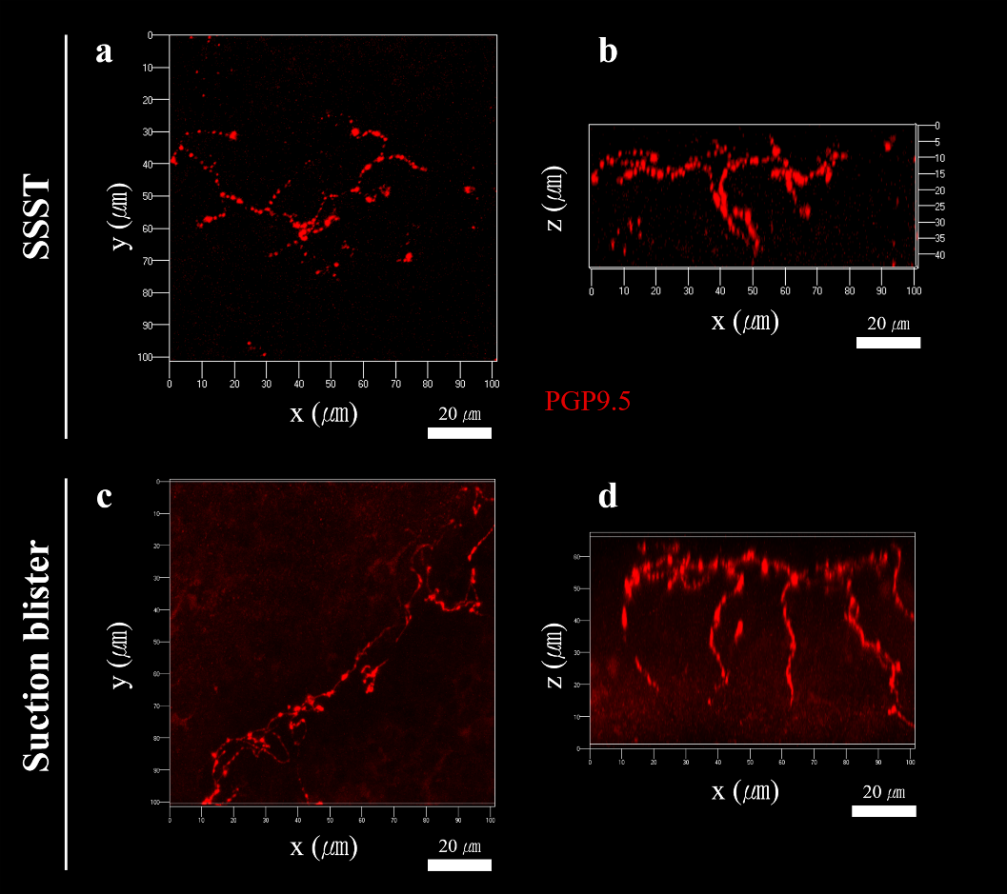


**Supplementary Figure 3.** Schematic aged-related morphological alteration of epidermal innervation. (a) Aged people showed decreased numbers and simplified structures of intraepidermal nerve fibers compared with the younger subjects. The pattern dominant structural simplification appeard to proceed from a significant decrease in numbers. (b) The meaningful emergence and increase of vacant areas without the physical reach of epidermal innervation became more prevalent according to natural aging.


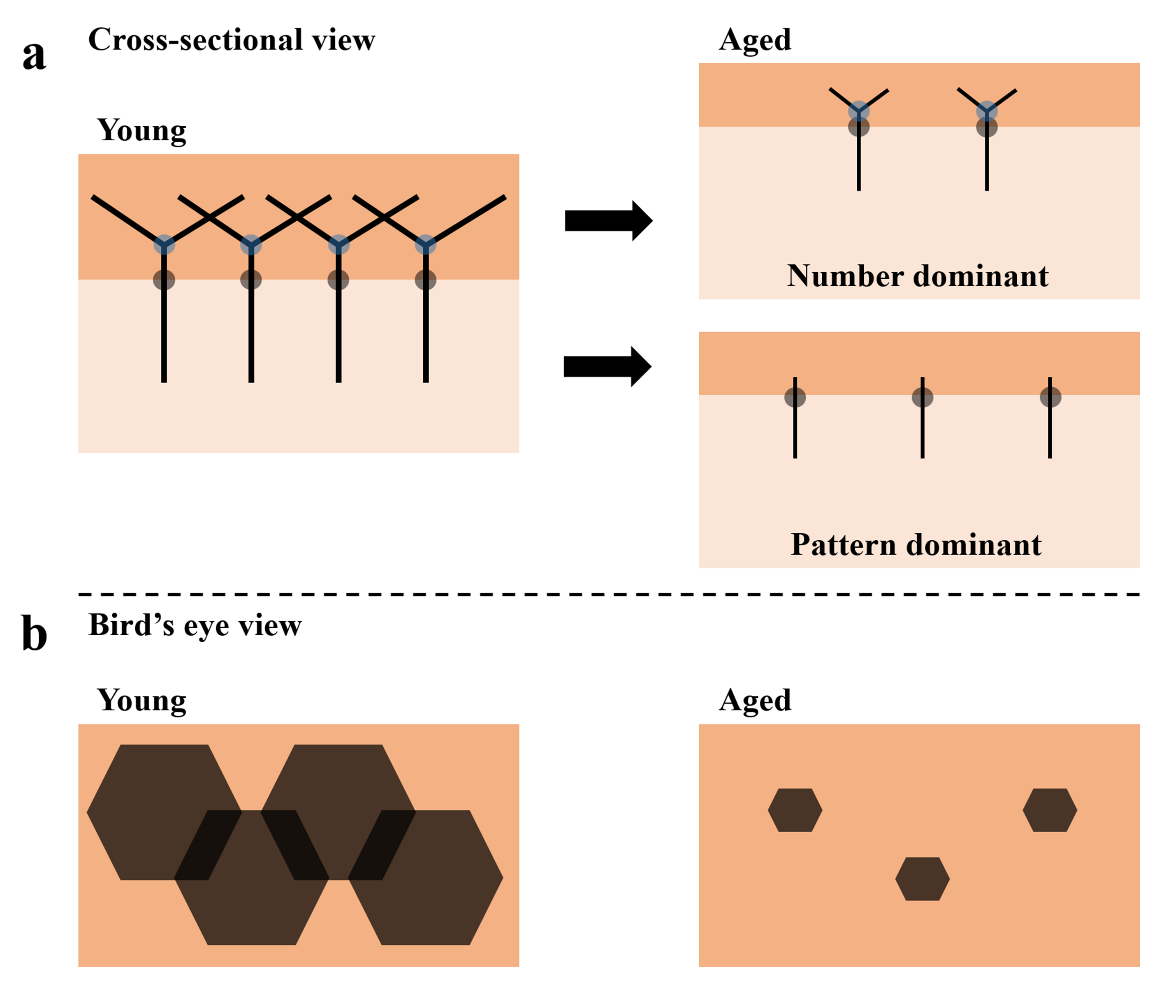


**Supplementary movie**

**Supplementary Movie. 1.** A 3-D view of the intraepidermal nerve fibers (IENFs) imaged from healthy skin samples provided a dense, intermingled network of cutaneous nerves in a more expanded area compared with the results from the conventional section-based method. PGP9.5-labeled IENFs (red) and nuclear stained signals (blue) are shown in the volume image. The 3-D image was obtained with a 40× HC PL APO CS2 40×/1.30 lens (z-stack step: 0.35㎛) and showed xyz-dimension of 1023.51, 828.96, and 113.50㎛, respectively. The 3-D image of IENFs was post-processed for making the movie using LAS X.
